# Systematic Analysis of Stress Granule Regulators-Associated Molecular Subtypes Predicts Drug Response, Immune Response, and Prognosis in Non-Small Cell Lung Cancer

**DOI:** 10.3389/fcell.2022.868918

**Published:** 2022-03-30

**Authors:** Dan Wang, Jiangen Ao, Youwen Xiong, Xinyi Zhang, Weifang Zhang

**Affiliations:** ^1^ Department of Pharmacy, The Second Affiliated Hospital of Nanchang University, Nanchang, China; ^2^ State Key Laboratory of Genetic Engineering, Shanghai Engineering Research Center of Industrial Microorganisms, School of Life Science, Fudan University, Shanghai, China; ^3^ Department of Testing, Jiangxi Center of Medical Device Testing, Nanchang, China; ^4^ Department of Respiratory Diseases, The Second Affiliated Hospital of Nanchang University, Nanchang, China

**Keywords:** non-small cell lung cancer, stress granule, molecular subtypes, drug response, immune response, prognosis

## Abstract

Lung cancer has the world’s second highest cancer incidence and second highest cancer-related mortality rate. However, the mechanism underlying non-small cell lung cancer (NSCLC) remained to be unclear. Overall, this study for the first time revealed Stress Granule Regulators were mutated and dysregulated in NSCLC samples by analyzing TCGA database. Moreover, three subtypes of NSCLC were identified based on the expression levels of Stress Granule Regulators. Patients in cluster 2 showed a higher survival rate than those in clusters 1 and 3. Bioinformatics analysis indicated the cell cycle, mTOR signaling pathway, EGFR signaling, PI3K/Akt signaling and DNA damage repair signaling were significantly related to molecular subtypes. Moreover, we performed a prediction analysis of the response to the inhibitors against the aforementioned signaling. Our results showed patients in C2 NSCLC had the highest sensitivity to MK.2206 (AKT.inhibitor) and Rapamycin (mTOR inhibitor). Patients in C3 NSCLC had the highest sensitivity for Temsirolimus (PI3K/mTOR signaling), BIBW2992 (EGFR signaling), Erlotinib (EGFR signaling), PD.0332991 (CDK4/6 inhibitor), CGP.60474 (CDK inhibitor), and Gefitinib (EGFR signaling). Moreover, our results showed patients in C1 NSCLC had the highest sensitivity to AKT.inhibitor, AZD6482 (PI3K inhibitor). To evaluate the response to immune therapy of different subtypes, we analyzed the tumor immune inflation, immune regulators expression, and TIDE score in different SG related subtypes. These results showed that C2 and C3 may be more sensitive to immune therapy. To better predict the prognosis of NSCLC, we analyzed the correlation between stress granule regulator expression and overall survival time in NSCLC and constructed a Stress Granule Score including EIF2S1, CTSG, EIF4G1, IGF2BP1, PABPC1 to predict the prognosis of NSCLC. Overall, this study for the first time uncovers the effect of stress particles on drug response, immune response, and prognosis, laying a new theoretical foundation for the NSCLC prognosis and treatment.

## Introduction

Lung cancer has the world’s second highest cancer incidence and second highest cancer-related mortality rate (Barta et al., 2019; Miranda-Filho et al., 2019). After an initial diagnosis of NSCLC (non-small cell lung cancer), the overall 5-year survival rate is less than 15% (Barta et al., 2019; Miranda-Filho et al., 2019). In the United States in 2021, there were 235,760 lung cancer cases (Siegel et al., 2021). Non-small cell lung cancer accounts for about 84 percent of all lung cancer cases (Brown et al., 2013). Adenocarcinoma, squamous cell carcinoma, and big cell carcinoma of the lung are the three primary histological subtypes of NSCLC (Travis, 2011). Chemotherapy is used on patients with stage II and higher NSCLC. Additional radiation was given to patients in stages III and IV. Atezolizumab was recently cleared by the FDA for use in stage II and stage III cancers. Despite tremendous advances in the treatment of NSCLC, there is still a need to investigate effective, tailored therapy options.

When cells are subjected to external stressors such as hypoxia, oxidative stress, arsenic acid virus infection, and so on, mRNA translation stops and stress granules (SR) are formed, which are found in the cytoplasm to protect cells and help cells adapt to their surroundings (Wang et al., 2020). When the ambient stress is removed, the stress particles depolymerize, release mRNA and protein, and continue cellular translation. At the start of translation, the SG is composed of mRNA as well as various translation initiation factors and RNA-binding proteins. The development of SG has numerous advantages for cell biology, including reduced energy consumption, prevention of protein production, and improved cell survival (Cao et al., 2020). Many stimuli induce the formation of SGs, and the formation of SGs is a defensive strategy used by cells to respond to their surroundings. Stress particles can help cells survive by sequestering pro-apoptotic factors (Lasfargues et al., 2012). Cell survival is linked to SG formation, and it has the ability to trap pro-apoptotic molecules. TRAF2 is the first apoptotic factor discovered to be linked to stress granules (Vanderweyde et al., 2013). According to recent research, RACK1 and ROCK1, the primary components that drive the p38/JNK apoptotic signaling cascade, are found in SGs and are beneficial to cell survival (Wang et al., 2020). Stress particles can alter the location of pro-apoptotic factors, thereby affecting their function. Of note, stress granules were also related to the NSCLC progression. For example, G3BP1, a key SG regulators, were found to be significantly overexpressed in NSCLC (Zheng et al., 2019). G3BP1 Depletion Increases Radiosensitisation by Inducing Oxidative Stress in Response to DNA Damage in NSCLC (Cho et al., 2019).

The researchers analyzed 503 NSCLC samples from the TCGA database and discovered three subtypes. In terms of patient prognosis, treatment responsiveness, and immune cell infiltration, we discovered that these three subgroups were very inconsistent. For that purpose, we devised a scoring system that allowed us to quantify the number of stress particles in individual patients and examine the link between stress particles and biological parameters. According to the findings, stress particles were linked to several cancer indicators, immunological infiltration, treatment sensitivity, and patient survival, according to the findings. This is the first study to thoroughly uncover the critical function of stress particles in NSCLC, laying a new theoretical foundation for the disease’s molecular process and treatment.

## Materials and Methods

### Data Collection

TCGA (https://portal.gdc.gov/Cancer.gov) database was used to get genome-wide expression data. The Molecular Signature Database was used to obtain a database of stress- granules-related genes (SGRGs) (MSigDB) (Liberzon et al., 2011; Liberzon et al., 2015).

### Identification of Differentially Expressed Genes

Furthermore, the limma R package was used to identify DEGs in order to acquire putative key genes in BMs. DEGs were screened using the following criteria: |fold change| > 1.5 and a *p*-value of less than 0.05.

### Identification of Stress Granule Subtypes in NSCLC

First, using univariate Cox regression (*p* 0.01), 138 DEGs were chosen for further investigation. These 138 genes have been labeled as “subtype-specific genes.”

Then, using the R package “ConsensusClusterPlus,” we performed unsupervised clustering of all the data using the expression of stress granule regulators (Wilkerson and Hayes, 2010).

### Pathway Enrichment Analysis

The DAVID database (https://david.ncifcrf.gov/) was used for the pathway enrichment analysis. An adjusted *p* value of 0.05 was considered statistically significant.

### Estimation of Immune Cell Infiltration

To measure the relative infiltration of 28 immune cell types in the tumor microenvironment, we used the CIBERSORT algorithm to analyze the amount of tumor immune infiltration in stress granule subtypes of NSCLC.

### Unsupervised Consensus Clustering

In this study, Unsupervised consensus clustering was applied to construct consensus matrix and subtyping samples based on the expression of Stress Granule Regulators using ConsensusClusterPlus R package.

### Generation of a Stress Granule-Dependent Risk Model

A stress particle-related risk score was created using principal component analysis (PCA), which was defined as the stress granule score:

Stress Granule Score=(PC1i+PC2i).

The optimal cutoff values derived by the survminer R program were used to separate NSCLC samples into high and low stress particle score groups. To further examine the model’s predictive performance, we ran Kaplan-Meier analyses of stress particle scores in NSCLC and generated hazard ratios (HRs) using univariate and multivariate Cox regression analysis. We used ROC curves to fit the Stress Granule score to significant clinical measures acquired through multivariate Cox regression and to assess the Stress Granule score’s accuracy. Logistic regression analysis of multivariate fit was performed using the R program “glmnet,” and the ROC curve was plotted using the R package “timeROC”.

We applied correlation analysis to discover the relationship between stress particle scores and 28 immune cell types in order to study the link between stress particle scores and biological parameters.

### Immunotherapy and Chemotherapy Response With Stress Granule Scores

Based on the publicly available pharmacogenomics database GDSC (Yang et al., 2013), we predicted the chemotherapeutic response for each sample. The half-maximal inhibitory concentration (IC50) of the samples was calculated using the R package “pRRophetic” (Geeleher et al., 2014).

## Results

### Analysis of Stress Granule Regulators Mutation and Expression Patterns in NSCLC

**FIGURE 1:**
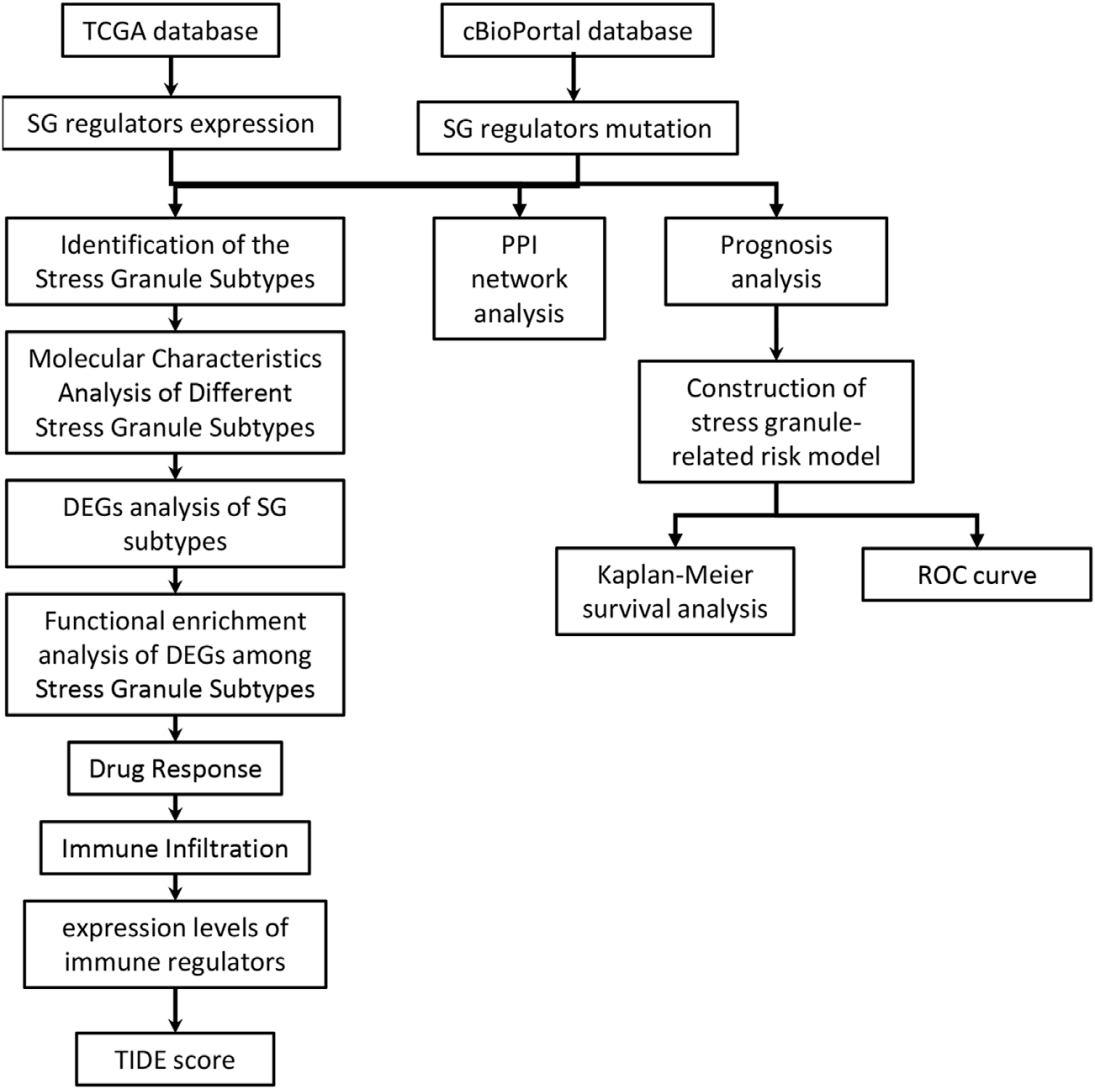
The workflow for the analysis of this study was presented in figure.

**FIGURE 2:**
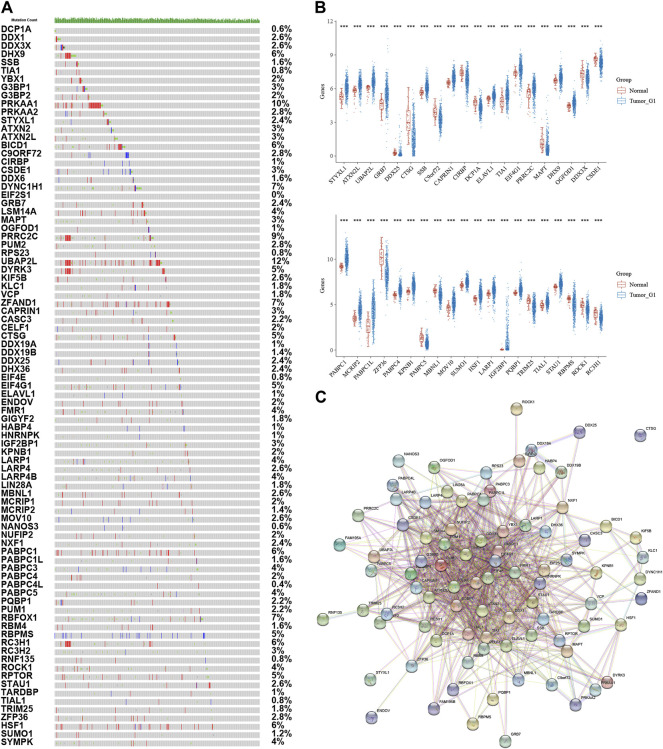
Analysis of Stress Granule Regulators Mutation and Expression Patterns in NSCLC. **(A)** genetic variants of stress granule regulators in NSCLC samples using Cbiopotal database. **(B)** Stress granule regulators were differently expressed in NSCLC samples. **(C)** Protein–protein interaction networks revealed that these stress granule-related proteins commonly interacted with one another.

The purpose of this study was to look at the roles of stress granule regulators in NSCLC. The workflow for the analysis of this study was presented in Figure 1. The cBioPortal database was used to look at the genetic variants of stress granule regulators in 1,680 instances (Figure 2A). UBAP2L, PRKAA1, PRRC2C, DYNC1H1, ZFAND1, RBFOX1, DHX9, BICD1, PABPC1, RC3H1, HSF1, DYRK3, CTSG, EIF4G1, RBPMS, and RPTOR were among the 87 stress granule regulators with varied degrees of genetic diversity. As shown in Figure 1A, the majority of stress granule regulators were amplified, deleted, or mutated in NSCLC, with UBAP2L having the highest rate of occurrence (12 percent).

Also, we analyzed the expression patterns of 87 stress granule regulators in NSCLC using TCGA databases. In NSCLC samples, STYXL1, ATXN2L, UBAP2L, GRB7, DDX25, CTSG, SSB, CAPRIN1, CIRBP, DCP1A, ELAVL1, TIA1, EIF4G1, PRRC2C, MAPT, DHX9, OGFOD1, DDX3X, CSDE1, HABP4, PRKAA2, CELF1, DYRK3, DDX1 were significantly dysregulated in NSCLC. These results demonstrated that stress granule regulators have a crucial role in NSCLC (Figure 2B).

Furthermore, protein–protein interaction networks revealed that these stress granule-related proteins commonly interacted with one another (Figure 2C). Several hub SG regulators were identified, including ATXN2, DDX6, DDX3X, DHX9, EIF4G1, CAPRIN1, ATXN2L, EIF4E, ELAVL1, G3BP1.

### Identification of the Stress Granule Subtypes in NSCLC

**FIGURE 3:**
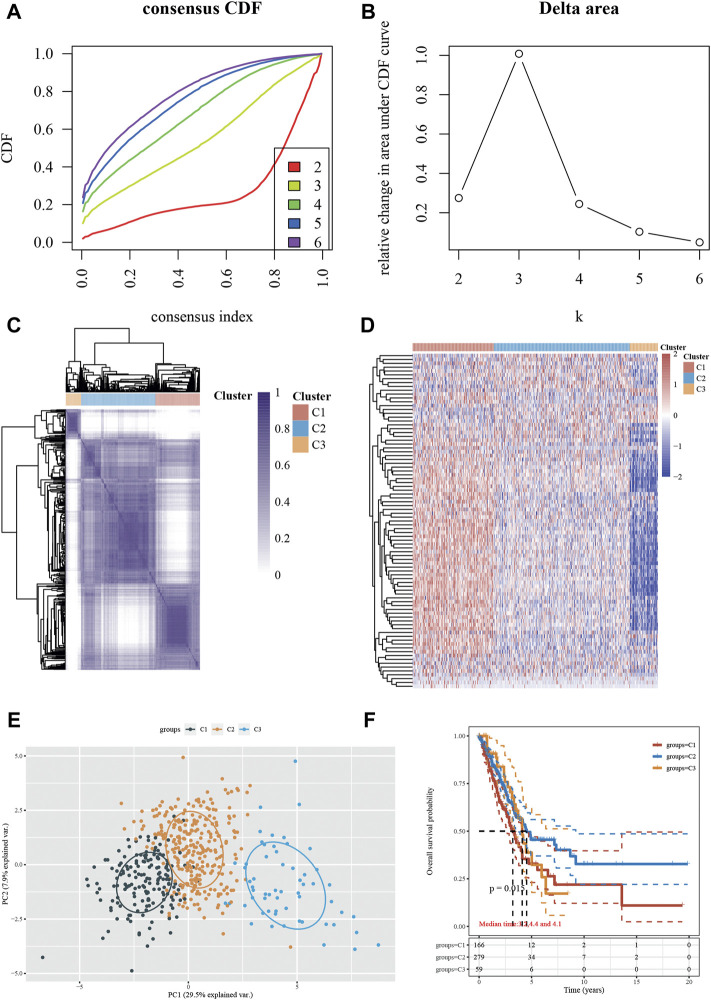
Identification of the Stress Granule Subtypes in NSCLC. **(A–C)** Unsupervised clustering of the expression of 60 stress granule-related genes divided all NSCLC cases into three groups (SGRGs). **(D)** Heatmap analysis showed the expression of stress granule-related genes in 3 clusters. **(E)** PCA analysis of all samples in three clusters. **(F)** Kaplan-Meier curves of overall survival for patients with NSCLC in three subtypes.

For the current investigation, a total of 503 NSCLC samples were analyzed, along with complete survival data. We identified 87 differentially expressed genes (DEGs) within clusters using univariate Cox regression analysis to create a Stress Granule subtype with prognostic significance. Following that, we performed secondary clustering, which revealed that the optimal number of clusters was 3, as determined by CDF curves (Figures 3A,B). Unsupervised clustering of the expression of 60 stress granule-related genes separated all NSCLC cases into three groups (SGRGs) (Figure 3C). In cluster 1, the majority of SGRGs were highly up-regulated, while in cluster 3, they were dramatically down-regulated (Figure 3D). The three clusters had different prognoses, according to the survival analysis. Patients in cluster 2 showed a higher survival rate than those in clusters 1 and 3 (Figures 3E,F).

### Molecular Characteristics Analysis of Different Stress Granule Subtypes

**FIGURE 4:**
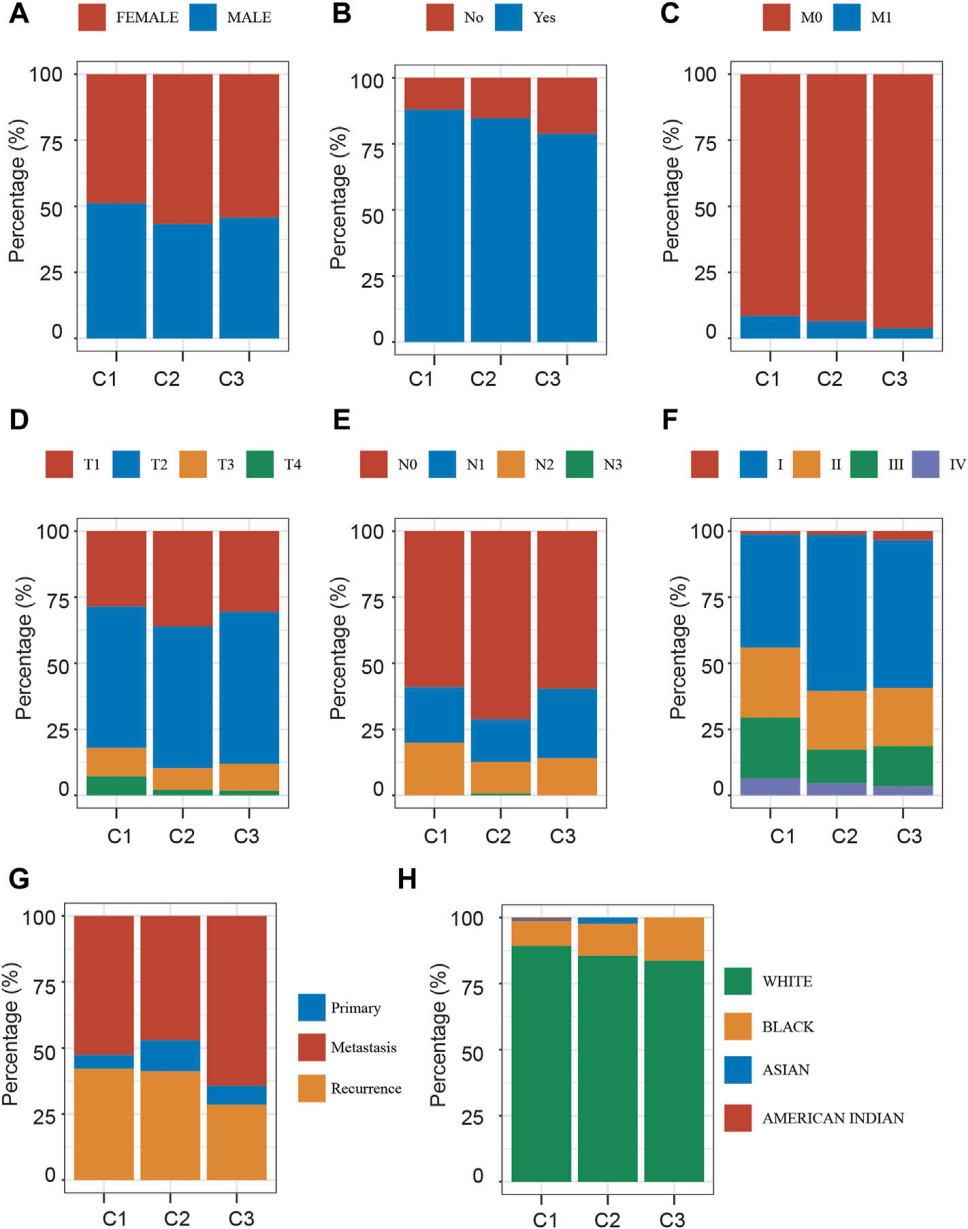
Clinical Characteristics Analysis of Different Stress Granule Subtypes. **(A)** The distribution of gender in Different Stress Granule Related Subtypes of NSCLC was shown. **(B)** The distribution of smoking status in Different Stress Granule related Subtypes of NSCLC was shown. **(C)** The distribution of M stage in Different Stress Granule related Subtypes of NSCLC was shown. **(D)** The distribution of T stage in Different Stress Granule related Subtypes of NSCLC was shown. **(E)** The distribution of N stage in Different Stress Granule related Subtypes of NSCLC was shown. **(F)** The distribution of grade in Different Stress Granule related Subtypes of NSCLC was shown. **(G)** The distribution of metastasis status in Different Stress Granule related Subtypes of NSCLC was shown. **(H)** The distribution of race in Different Stress Granule related Subtypes of NSCLC was shown.

Furthermore, we compared the three NSCLC subtypes in terms of gender, smoking status, M stage, T stage, N stage, grade, metastasis status, and race (Figures 4A–H). Clinically, we found that the C2 subtype has a disproportionately high proportion of female patients (Figure 4A). The C1 subtype has the largest percentage of smokers (Figure 4B). The incidence of Stage II, Stage III, and Stage IV was similar in groups C2 and C3, but substantially greater in groups C3 and C4. In comparison to C1 and C4, groups C2 and C3 had a greater percentage of T3 and T4and a substantially lower percentage of N0. The rate of metastatic spread at diagnosis was not significantly different among the four groups (*p* = 0.762).

### DEGs Analysis of Different Stress Granule Subtypes in NSCLC

**FIGURE 5:**
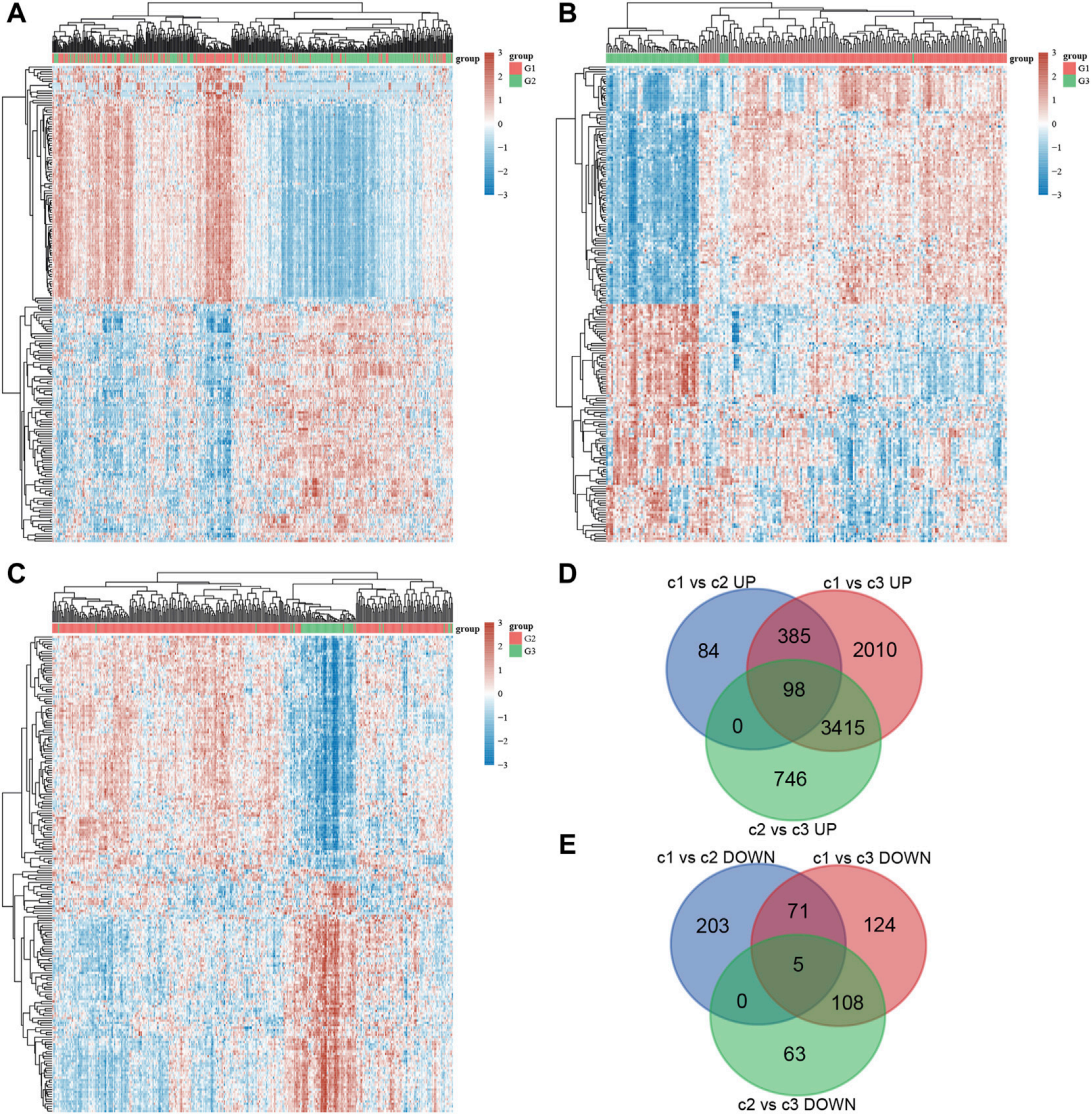
DEGs analysis of different stress granule subtypes in NSCLC. **(A)** Heatmap analysis showed DEGs between subtype 1 and subtype 2 of NSCLC. **(B)** Heatmap analysis showed DEGs between subtype 1 and subtype 3 of NSCLC. **(C)** Heatmap analysis showed DEGs between subtype 2 and subtype 3 of NSCLC. **(D,E)** Venn map analysis of DEGs analysis in different stress granule subtypes of NSCLC.

In order to understand the potential mechanisms underlying the effect of stress granules on NSCLC progression, we analyzed the DEGs between different stress granule subtypes in NSCLC. In total, 913 DEGs, including 476 upregulated and 437 downregulated genes, were identified in cluster1 compared to cluster2 NSCLC samples (Figure 5A). 913 DEGs, including 476 upregulated and 437 downregulated genes, were identified in cluster1 compared to cluster3 NSCLC samples (Figure 5B). 913 DEGs, including 476 upregulated and 437 downregulated genes, were identified in cluster2 compared to cluster3 NSCLC samples (Figure 5C). Among them, only 98 up-regulated and five suppressed genes in all types were revealed to be subtype specific and expressed (Figures 5D,E).

### Functional Enrichment Analysis of DEGs of Stress Granule Subtypes in NSCLC

**FIGURE 6:**
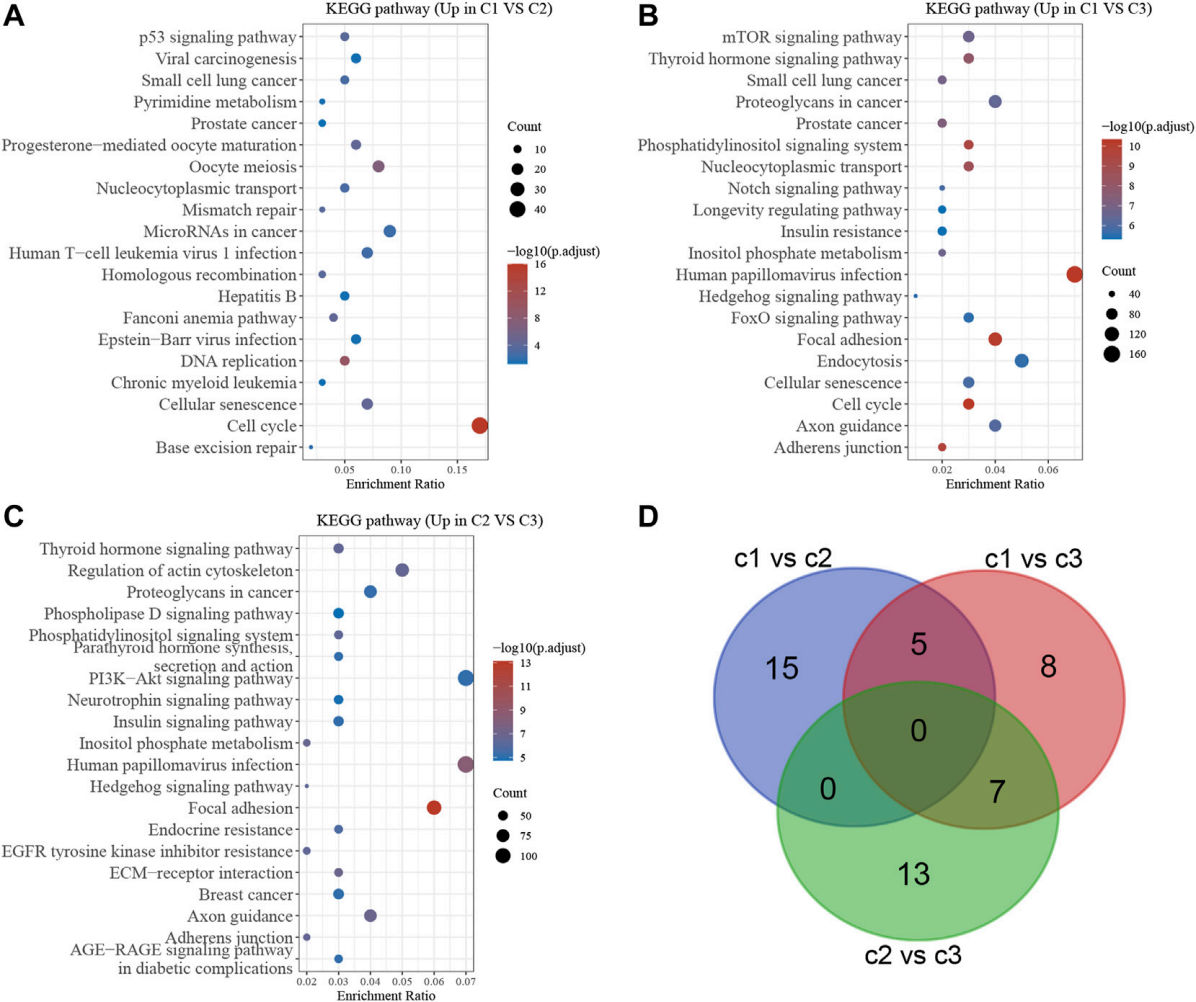
Bioinformatics analysis of DEGs in different stress granule subtypes of NSCLC. **(A)** Bioinformatics analysis of DEGs between subtype 1 and subtype 2 in NSCLC. **(B)** Bioinformatics analysis of DEGs between subtype 1 and subtype 3 of NSCLC. **(C)** Bioinformatics analysis of DEGs between subtype 2 and subtype 3 of NSCLC. **(D)** Venn map analysis showed stress granule related signaling of NSCLC.

To further determine the biological functions of the DEGs, KEGG enrichment analysis was performed using DAVID. As shown in Figure 6, the up-regulated genes in Cluster 1 were significantly related to base excision repair, cell cycle, cellular senescence, chronic myeloid leukemia, DNA replication, EpsteinBarr virus infection, Fanconi anemia pathway, Hepatitis B, Homologous recombination, human T cell leukemia virus infection, MicroRNAs in cancer Mismatch repair Nucleocytoplasmic transport, Oocyte meiosis, and small cell lung cancer are all issues that need to be addressed (Figure 6A). Meanwhile, the up-regulated genes in Cluster 2 were significantly related to the cell cycle, cellular senescence, Endocytosis, Focal adhesion, FoxO signaling, Hedgehog signaling, human papillomavirus infection, Inositol phosphate metabolism, Insulin resistance, Longevity-regulating pathway Notch signaling, Nucleocytoplasmic transport, Phosphatidylinositol signaling system, small cell lung cancer, thyroid hormone signaling, mTOR signaling (Figure 6B). Meanwhile, the up-regulated genes in Cluster 3 were significantly related to adherens junction, axon guidance, Breast cancer, EGFR tyrosine kinase inhibitor resistance, Hedgehog signaling, Human papillomavirus infection, Insulin signaling, Neurotrophin signaling, PI3KAkt signaling, Parathyroid hormone synthesis, secretion and action, Phosphatidylinositol signaling system, phospholipase D signaling (Figure 6C).

### The Molecular Subtypes of Stress Granule Regulators Predict Drug Response in Non-Small Cell Lung Cancer

**FIGURE 7:**
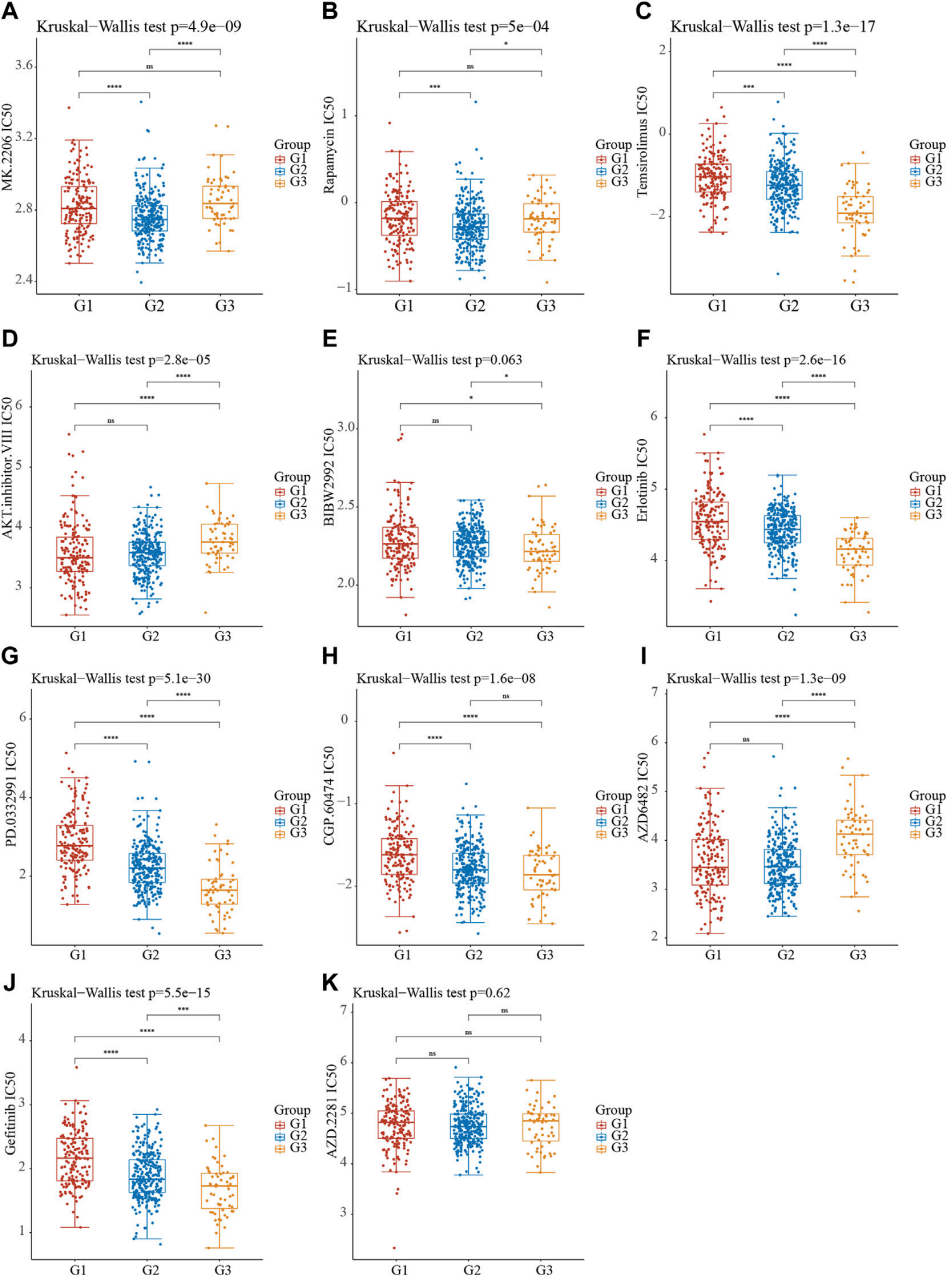
The Molecular Subtypes of Stress Granule Regulators Predict Drug Response in Non-Small Cell Lung Cancer. **(A–K)** Stress Granule Regulators based subtypes predicted the response to MK.2206 (AKT.inhibitor) **(A)**, Rapamycin (mTOR inhibitor) **(B)**, Temsirolimus (PI3K/MTOR signaling) **(C)**, AKT.inhibitor **(D)**, BIBW2992 (EGFR signaling) **(E)**, Erlotinib (EGFR signaling) **(F)**, PD.0332991 (CDK4/6 inhibitor) **(G)**, CGP.60474 (CDK inhibitor) (H), AZD6482 (PI3K inhibitor) **(I)**, Gefitinib (EGFR signaling) **(J)** and AZD2281 **(K)** in NSCLC.

The bioinformatics analysis showed the cell cycle, mTOR signaling pathway, EGFR signaling, PI3KAkt signaling and DNA damage repair signaling were significantly related to molecular subtypes (Figure 6D). Understanding the efficiency of the inhibitors against these signaling could provide the potential therapy choose for NSCLC. Using the pRRophetic R package, we performed a prediction analysis of the response to the inhibitors against the aforementioned signaling. Our results showed patients in C2 NSCLC had the highest sensitivity to MK.2206 (AKT inhibitor) and Rapamycin (mTOR inhibitor) with the lowest IC 50 value. Patients in C3 NSCLC had the highest sensitivity for Temsirolimus (PI3K/MTOR signaling), BIBW2992 (EGFR signaling), Erlotinib (EGFR signaling), PD.0332991 (CDK4/6 inhibitor), CGP.60474 (CDK inhibitor), and Gefitinib (EGFR signaling) with the lowest IC 50 value. Moreover, our results showed patients in C1 NSCLC had the highest sensitivity to AKT inhibitor, AZD6482 (PI3K inhibitor) (Figures 7A–K).

### The Difference in Immune Infiltration Between Stress Granule Subtypes

**FIGURE 8:**
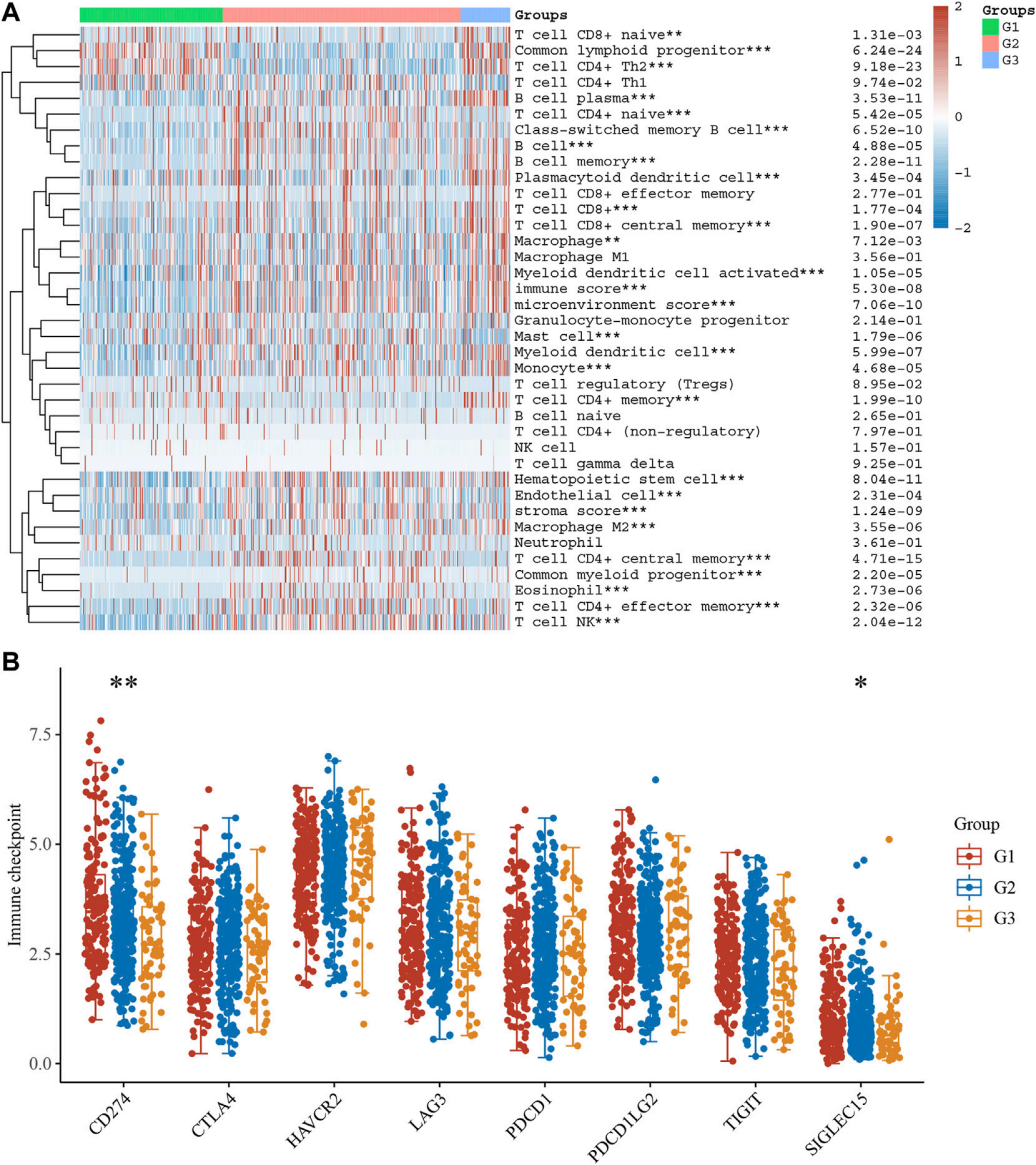
The Difference in Immune Infiltration Between Stress Granule Subtypes. **(A)** The Immune Infiltration levels of different Stress Granule related Subtypes in NSCLC were shown. **(B)** The expression levels of immune regulators among these subtypes of NSCLC were analyzed.

From the TCGA dataset, we created a heatmap to display the relative abundance of immune invading cell subpopulations (Figure 8A). According to our findings, immune cell infiltration differed significantly amongst the three subtypes. As can be observed, there are disparities in infiltration levels among human immune cells of stress granule subtypes.

As present in Figure 7A, T cell CD8^+^ nave, common lymphoid progenitor, and T cell CD4^+^ Th2 cells are highly enriched in the C1 and C3 subtypes of NSCLC. C2 had high inflation levels of T cell CD4^+^ nave, T cell CD4^+^ central memory, T cell CD4^+^ effector memory, and endothelial cells. However, C3 had high inflation levels of myeloid dendritic cells, T cell CD8^+^, T cell CD8^+^ central memory, Myeloid dendritic cell activated, hematopoietic stem cells, macrophages, macrophagage M1, macrophagage M2, B cell memory, T cell NK, plasmacytoid dendritic cell, and B cell plasma. Very interestingly, the immune score analysis showed immune score, stroma score, and microenvironment score were highest in C3 NSCLC samples and lowest in C1 NSCLC samples.

We also analyzed the expression levels of immune regulators among these subtypes of NSCLC. The results showed CD274 was significantly up-regulated in C1 compared to C2 and C3 (Figure 8B). These results showed that C2 and C3 may be more sensitive to immune therapy.

### Predictive ICB Response of Identified Stress Granule Subtypes in NSCLC

**FIGURE 9:**
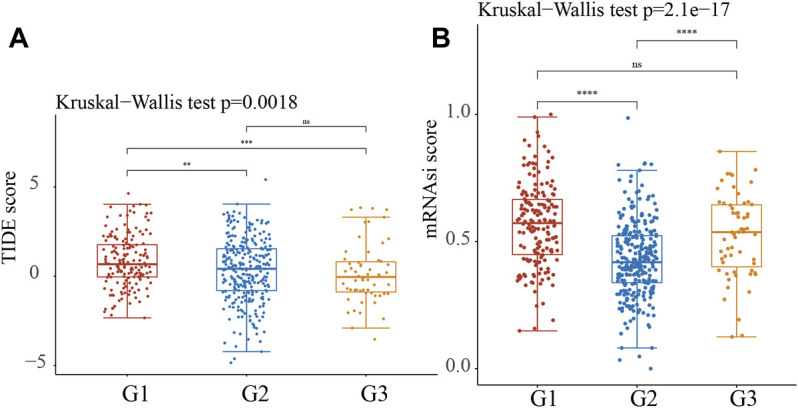
Predictive ICB response of identified Stress Granule Subtypes in NSCLC. **(A)** The TIDE score was calculated in the TCGA LUAD cohort to predict immune treatment response of Stress Granule Subtypes in NSCLC. **(B)** The mRNAsi score of Stress Granule Subtypes in NSCLC was calculated.

The TIDE score was calculated in the TCGA LUAD cohort to predict immune treatment response. According to the findings, the TIDE score was significantly lower in the C2 and C3 subtypes of NSCLC than in the C1 subtype of NSCLC (Figure 9A). According to these findings, patients with the C2 and C3 subtypes may be more sensitive to ICB therapy, as measured by the TIDE score.

### The Dysregulation of Stress Granule Regulators Was Related to the Poor Prognosis of NSCLC

**FIGURE 10:**
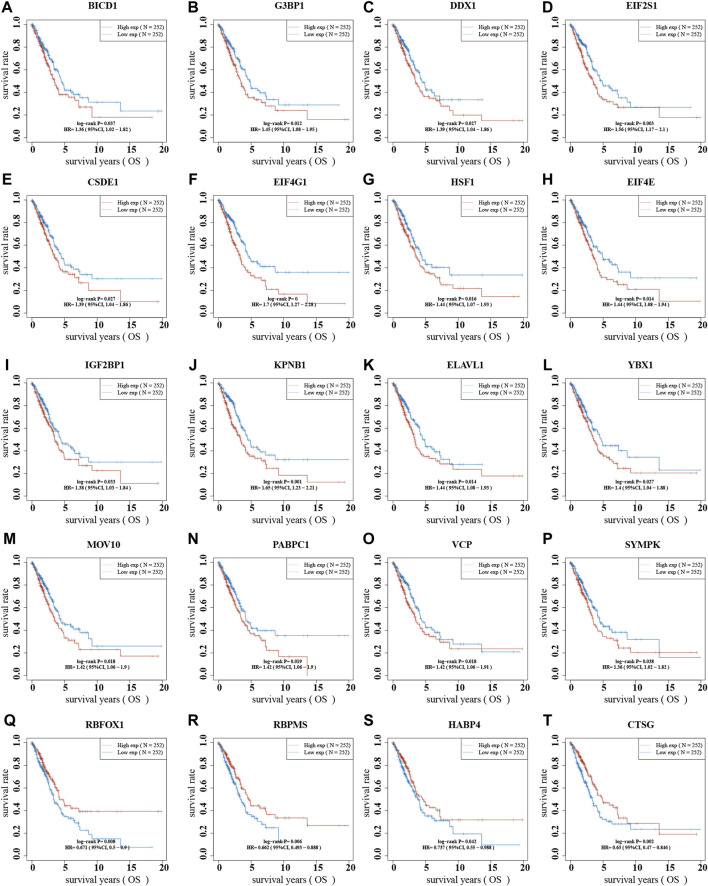
The dysregulation of stress granule regulators was related to the poor prognosis of NSCLC. **(A–T)** The correlation between stress granule regulator expression and overall survival time in NSCLC were analyzed, including SYMPK, BICD1, IGF2BP1, CSDE1, DDX1, YBX1, PABPC1, MOV10, VCP, HSF1, ELAVL1, SSB, EIF4E, G3BP1, EIF2S1, KPNB1, EIF4G1, CTSG, RBPMS, RBFOX1, and HABP4.

To better predict the prognosis of NSCLC, we analyzed the correlation between stress granule regulator expression and overall survival time in NSCLC. The higher levels of SYMPK, BICD1, IGF2BP1, CSDE1, DDX1, YBX1, PABPC1, MOV10, VCP, HSF1, ELAVL1, SSB, EIF4E, G3BP1, EIF2S1, KPNB1, EIF4G1 are related to shorter OS time in patients with NSCLC. However, higher levels of CTSG, RBPMS, RBFOX1, and HABP4 are linked to a prolonged OS duration in individuals with NSCLC (Figures 10A–D).

### Construction of Stress Granule-Related Risk Model

**FIGURE 11:**
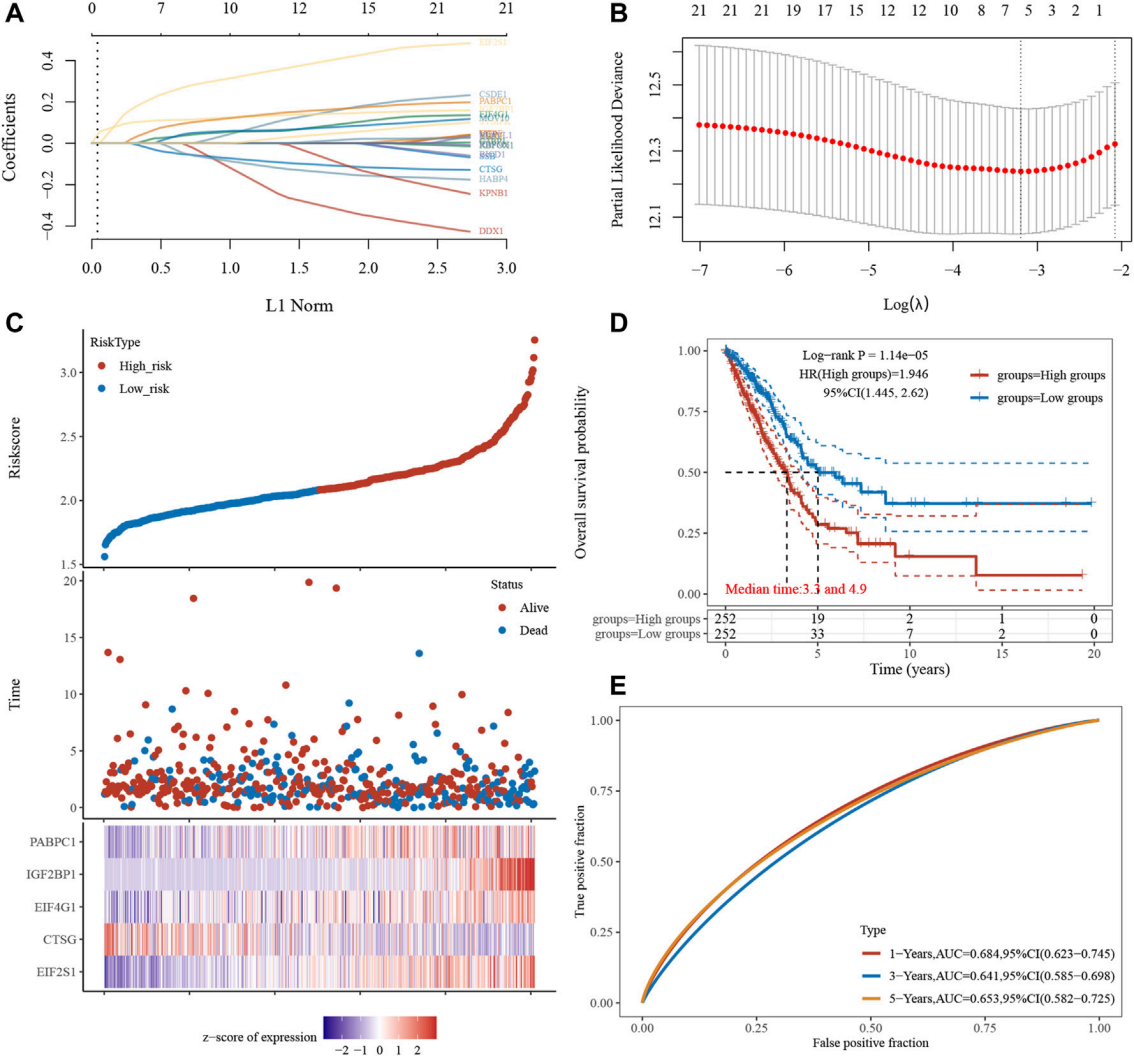
Risk Model Construction and Stress Granule Score. **(A)** LASSO coefficients profiles of Stress Granule regulators. **(B)** LASSO regression with tenfold cross-validation obtained five prognostic genes using minimum lambda value. **(C)** Individual patients with NSCLC had their stress granule levels quantified using the Stress Granule score, which took into account the individual variability and complexity of gene expression levels. **(D)** Kaplan-Meier survival analysis showed patients with low stress granule scores had a significant survival advantage. **(E)** ROC curve analysis of the Stress Granule risk model in NSCLC.

We created a to better predict prognosis and guide tailored treatment (Figures 11A,B). Individual patients with NSCLC had their stress granule levels quantified using the Stress Granule score, which took into account the individual variability and complexity of gene expression levels (Figure 11C). We next evaluate whether the Stress Granule Score could be used to predict the outcome of NSCLC patients. Using the survminer R program, the ideal cut-off value was determined, and patients were divided into high- and low-stress granule score groups. We used Kaplan-Meier survival analysis and univariate cox regression analysis to find that patients with low stress granule scores had a significant survival advantage (Figure 11D). According to the ROC curve, the Stress Granule score in our current study had a good predictive performance in terms of 1, 3, and 5 year AUC, with values of 0.68, 0.67, and 0.67, respectively (Figure 11E).

## Discussion

In conclusion, our research identifies three stress granule-associated NSCLC subgroups with distinct prognostic, gender, mutational, and immunoinfiltrating characteristics. To quantify stress particle levels in individual tumors, we built a risk model termed the stress particle score; the stress particle score demonstrated considerable advantages in predicting prognosis and immunotherapy response. Our research for the first time discovered the roles of stress granule associated molecular subtypes in prediction of drug response, immune response, and prognosis in NSCLC, laying a new theoretical foundation for the NSCLC prognosis and treatment.

In this study, we analyzed the expression and mutation pattern of stress granule regulators. We found the majority of stress granule regulators were amplified, deleted, or mutated in NSCLC, with UBAP2L was the most frequently mutated genes. Also, we revealed UBAP2L was significantly up-regulated in NSCLC. UBAP2L has been shown to be overexpressed in several tumors, such as liver carcinoma (Wang et al., 2017), breast cancer (He et al., 2018) and gastric cancer (Lin et al., 2021). Also, previous studies showed UBAP2L was amplified in LUAD and is critical for tumor metastasis (Aucagne et al., 2017). UBAP2L silencing suppressed the cellular proliferation and invasion of NSCLC. Our results, together with previous reports demonstrated UBAP2L was potential crucial regulator of NSCLC.At present, tissue typing alone can no longer meet the needs of individualized treatment of lung cancer. With the development of molecular biotechnology, molecular typing of lung cancers of different tissue subtypes is carried out, and individualized treatment of molecular targets is guided according to the mutation spectrum of lung cancer driver genes (Malone et al., 2020). Dr. Mark Kris reported the results of a genome-wide or multiple molecular typing study, which used a multiplex detection method to detect 10 known driver mutation genes in 830 lung adenocarcinoma tumor tissues. The results showed that 60% (252/422) of the tumor tissues had driver mutations, including 107 cases of KRAS (25%), 98 cases of EGFR (23%), 14 cases of ALK rearrangement (6%). Targeted drug therapy such as EGFR-TKI has demonstrated good clinical effects and has gradually become part of the clinical standard treatment of NSCLC, but it still faces optimal timing of targeted drug treatment, easy drug resistance, and the trade-off between the pros and cons of chemotherapy, etc (Goto et al., 2017; Lin et al., 2017; Nan et al., 2017). At present, irreversible ErbB family inhibitors against EGFR-TKI resistance, such as afatinib, have achieved good results in large-scale clinical trials and are expected to become part of a new generation of lung cancer targeted therapy drugs (Hoffknecht et al., 2015; Zhang et al., 2017). Today, new targeted drugs for molecular typing of lung cancer, such as crizotinib, are constantly on the market, injecting new vitality into molecular targeted therapy of lung cancer (Bang, 2011). The emergence of new lung cancer molecular subtypes of RET fusion type and ROS1 fusion type has brought new vitality to the establishment of a new molecular typing diagnosis and treatment model (Takeuchi et al., 2012; Kohno et al., 2015). In the present study, we for the first time showed NSCLC could be divided into 3 subtypes based on the expression of stress granule-related genes. Most of SGRGs were significantly up-regulated in cluster 1 compared to cluster 2 and cluster 3. Interestingly, we revealed Patients in cluster 2 had a longer survival timer than those in clusters 1 and 3, which would provide novel insights for NSCLC treatment based on Stress Granule regulators.

To further explore the biological functions of the Stress Granule regulators in NSCLC, we performed KEGG enrichment analysis of DEGs in different subtypes. The bioinformatics analysis showed the cell cycle, mTOR signaling pathway, EGFR signaling, PI3KAkt signaling and DNA damage repair signaling were significantly related to molecular subtypes. These signaling had been demonstrated to be crucial for NSCLC progression. For example, the signaling pathway PI3K/Akt/mTOR is an essential intracellular signaling pathway (Alzahrani, 2019). It affects the function of downstream effector molecules, which is critical for cell apoptosis and survival, and it is linked to the formation and progression of NSCLC and plays a critical role in treatment resistance (Gadgeel and Wozniak, 2013; Sun et al., 2015; Chen et al., 2020). PI3K/AKT inhibitors have been demonstrated to suppress tumor cell proliferation *in vitro* and drive tumor cells to join the apoptotic program (Gadgeel and Wozniak, 2013; Sun et al., 2015; Chen et al., 2020). The use of PI3K/AKT signaling pathway inhibitors in NSCLC with high AKT expression can improve chemotherapy-induced cell death. Drug-induced death of lung cancer cells can be improved by inhibiting the PI3K signaling. A number of PI3K, Akt, and mTOR inhibitors are developed and in early clinical trials in NSCLC, such as Pictilisib, PX-866, buparlisib, and pilaralisib (Tan, 2020). in a Phase IB clinical trial in NSCLC showed 29 (43.9%) of the 66 patients showed a partial response, whereas 20 (30.9%) maintained stable disease after treated with Pictilisib in combination with chemotherapy (Soria et al., 2017). In the unselected NSCLC population, the study of PX-866 in advanced refractory NSCLC found no significant improvement in PFS (Levy et al., 2014). However, after stopping docetaxel, one PIK3CA mutated NSCLC patient exhibited a prolonged response to PX-866 treatment (Levy et al., 2014). Pilaralisib was also tested as a monotherapy in a Phase I trial, showing limited responses in NSCLC patients. Despite many efforts were paid, the efficiency of PI3K inhibitors is limited.

Moreover, we performed a prediction analysis of the response to the inhibitors against the aforementioned signaling in different subtypes of NSCLC. Our results showed patients in C2 NSCLC had the highest sensitivity to MK.2206 (AKT.inhibitor) and Rapamycin (mTOR inhibitor) with the lowest IC 50 value. Patients in C3 NSCLC had the highest sensitivity for Temsirolimus (PI3K/mTOR signaling), BIBW2992 (EGFR signaling), Erlotinib (EGFR signaling), PD.0332991 (CDK4/6 inhibitor), CGP.60474 (CDK inhibitor), and Gefitinib (EGFR signaling) with the lowest IC 50 value. Moreover, our results showed patients in C1 NSCLC had the highest sensitivity to AKT.inhibitor, AZD6482 (PI3K inhibitor) (Figures 6A–K). The identification of EGFR-activating mutations as predictive biomarkers for first-generation EGFR tyrosine kinase inhibitors (TKIs) has ushered in a new age of precision oncology for the treatment of advanced EGFR-mutated NSCLC (Chalmers et al., 2019; Liang et al., 2019; Wang et al., 2019). Despite the first-and second-generation EGFR TKIs’ robust effectiveness, disease recurrence is unavoidable. Because the EGFR T790M mutation is a key source of disease recurrence, third-generation irreversible EGFR inhibitors targeted to target EGFR T790M and activating mutations have shown promising clinical effectiveness and safety (Cross et al., 2014). Unfortunately, disease development is unavoidable, and a variety of resistance mechanisms have been identified, but there are few options for further treatment. One of the features of malignant tumors that can proliferate indefinitely is faulty cell cycle regulation. CDK4/6 inhibitors can stop tumor cells from progressing from S to G phase, halting tumor growth (Pernas et al., 2018). CDK4/6 inhibitors have been shown to be effective In breast cancer (Pernas et al., 2018). Cell cycle regulation is frequently abnormal in NSCLC, occurring in approximately 22–45% of cases, implying that CDK4/6 inhibitors may be effective in this disease (Wikman and Kettunen, 2006). 8/16 (50 percent) of patients had stable disease after Palbociclib treatment in a phase II NSCLC clinical trial. In a Phase II Practical Basket study, anticancer efficacy was observed in patients with CDKN2A-altered NSCLC.

In recent years, immunotherapy represented by immune checkpoint inhibitors has significantly improved the survival of patients with advanced lung cancer and changed the treatment mode for lung cancer (D'Andrea and Reddy, 2020). With the development of immunotherapy, a number of phase III clinical studies have confirmed that the efficacy of ICIs alone is superior to chemotherapy (Saxena et al., 2020). Pembrolizumab has been approved by the FDA as a first-line therapy NSCLC who are PD-L1 positive (TPS 50%) (Berardi, 2019; Nosaki et al., 2019). In KEYNOTE-024 clinical trial, and the pembrolizumab-treated group had a higher ORR and a lower frequency of treatment-related adverse events than the chemotherapy group (Berardi, 2019; Nosaki et al., 2019). In the KEYNOTE-010 study, the NSCLC after pembrolizumab treatment had longer OS than chemotherapy (Berardi, 2019; Nosaki et al., 2019). In this study, we also perform analysis to predict the immune therapy response for different Stress Granule based subtypes. The results showed CD274 was significantly up-regulated in C1 compared to C2 and C3. Moreover, the TIDE score was significantly lower in the C2 and C3 subtypes of NSCLC than in the C1 subtype of NSCLC. These findings, patients with the C2 and C3 subtypes may be more sensitive to ICB therapy, as measured by the TIDE score.

A recently published meta-analysis suggested that high levels of intratumoral and interstitial CD8^+^ T cells can predict the efficacy of immunotherapy in multiple tumor types, including immunotherapy alone and immunocombination therapy (Li et al., 2021). A phase II study of NSCLC observed that ipilimumab neoadjuvant therapy had significant CD28-dependent activation of both CD4^+^ T cells and CD8^+^ T cells (Yi et al., 2017). Bruni et al. (2020) summarized and analyzed 17 kinds of immune cells that are meaningful for tumor prognosis, and found that the predictive value of immune components is related to tumor type, the number, distribution and activity of immune cells. The results of the NEOSTAR study showed that neoadjuvant immunotherapy in the dual-ICI combination group could induce the proliferation of local CD3^+^ T cells in the tumor, and the T cell diversity and memory T cells also increased significantly (Cascone et al., 2021). TILs can be explored not only as a biomarker but also as a potential tumor therapy.

To better predict the prognosis of NSCLC, we analyzed the correlation between stress granule regulator expression and overall survival time in NSCLC. The higher levels of SYMPK, BICD1, IGF2BP1, CSDE1, DDX1, YBX1, PABPC1, MOV10, VCP, HSF1, ELAVL1, SSB, EIF4E, G3BP1, EIF2S1, KPNB1, EIF4G1 are related to shorter OS time in patients with NSCLC. However, higher levels of CTSG, RBPMS, RBFOX1, and HABP4 are linked to a prolonged OS duration in individuals with NSCLC. Moreover, we constructed a Stress Granule Score including EIF2S1, CTSG, EIF4G1, IGF2BP1, PABPC1 to predict the prognosis of NSCLC. The results showed patients with low stress granule scores had a significant survival advantage. Among these prognosis related stress granule regulators, EIF4G1, IGF2BP1 had been demonstrated to be key regulators in cancer cells. For example, the IGF2 mRNA-binding proteins regulate mRNA cytoplasmic fate. Among them, IGF2BP1 has the most conserved “oncogenic” role in tumor cells, which induces a phenotype in mesenchymal tumor cells that includes altered actin dynamics, migration, invasion, proliferation, self-renewal, and anoikis resistance (Muller et al., 2019). In a variety of human malignancies, IGF2BP1 expression is linked to a bad prognosis. The disruption of mRNA degradation is the primary cause of IGF2BP1’s “carcinogenic” impact. IGF2BP1 inhibits endonuclease degradation of target transcripts, such as MYC mRNA, by binding to its target mRNA (Bell et al., 2013; Huang et al., 2018). EIF4F complex has been proven to play a key role in carcinogenesis throughout the last 2 decades (Chen et al., 2019). The EIF4G1 protein functions as a scaffold and binds to numerous other initiation factors, including EIF4E and EIF4AInteract, to help initiate cap-dependent translation in mammalian cells by attracting ribosomes to the capped ends of mRNA. Despite the fact that EIF4G1 is overexpressed in a number of malignancies, little is known about its role in the pathogenesis of NSCLC. Novel anti-cancer therapies that target components of the protein production apparatus are expected to overcome intratumoral heterogeneity.

Several limitations should be noted. First, the expression pattern of SG regulators were not confirmed using clinical NSCLC samples with western blot and Immunohistochemistry methods. Secondly, more validation using clinical inhibitors will strength the findings of this study. Thirdly, gain/loss of functional assays are needed to further confirm the molecular functions of SG regulators in NSCLC.

Overall, this study for the first time revealed Stress Granule Regulators were mutated and dysregulated in NSCLC samples by analyzing TCGA database. Moreover, three subtypes of NSCLC were identified based on the expression levels of Stress Granule Regulators. Patients in cluster 2 showed a higher survival rate than those in clusters 1 and 3. Bioinformatics analysis indicated the cell cycle, mTOR signaling pathway, EGFR signaling, PI3KAkt signaling and DNA damage repair signaling were significantly related to molecular subtypes. Moreover, we performed a prediction analysis of the response to the inhibitors against the aforementioned signaling. Our results showed patients in C2 NSCLC had the highest sensitivity to MK.2206 (AKT inhibitor) and Rapamycin (mTOR inhibitor). Patients in C3 NSCLC had the highest sensitivity for Temsirolimus (PI3K/mTOR signaling), BIBW2992 (EGFR signaling), Erlotinib (EGFR signaling), PD.0332991 (CDK4/6 inhibitor), CGP.60474 (CDK inhibitor), and Gefitinib (EGFR signaling). Moreover, our results showed patients in C1 NSCLC had the highest sensitivity to AKT inhibitor, AZD6482 (PI3K inhibitor). To evaluate the response to immune therapy of different subtypes, we analyzed the tumor immune inflation, immune regulators expression, and TIDE score in different SG related subtypes. These results showed that C2 and C3 may be more sensitive to immune therapy. To better predict the prognosis of NSCLC, we analyzed the correlation between stress granule regulator expression and overall survival time in NSCLC and constructed a Stress Granule Score including EIF2S1, CTSG, EIF4G1, IGF2BP1, PABPC1 to predict the prognosis of NSCLC. Overall, This study for the first time uncover the effect of stress particles on drug response, immune response, and prognosis, laying a new theoretical foundation for the NSCLC prognosis and treatment.

## Data Availability

The datasets presented in this study can be found in online repositories. The names of the repository/repositories and accession number(s) can be found in the article/Supplementary Material.
